# Impact of ultrasound examination shortly after kidney transplantation

**DOI:** 10.1007/s10353-017-0467-z

**Published:** 2017-02-16

**Authors:** Christoph Schwarz, Jakob Mühlbacher, Georg A. Böhmig, Marin Purtic, Eleonore Pablik, Lukas Unger, Ivan Kristo, Thomas Soliman, Gabriela A. Berlakovich

**Affiliations:** 1 Department of Surgery/Division of Transplantation, Medical University Vienna, Waehringer Guertel 18–20, 1090 Vienna, Austria; 2 Department of Medicine III/Division of Nephrology and Dialysis, Medical University Vienna, Vienna, Austria; 3 CeMSIIS, Section for Medical Statistics, Medical University Vienna, Vienna, Austria

**Keywords:** Ultrasound, Kidney transplantation, Outcome, Complications, Vascular

## Abstract

**Background:**

Ultrasound is routinely performed at our transplant unit within the first 48 h of kidney transplantation (KTX). The objective of this study was to evaluate the association of ultrasound results and, in particular, elevated resistance indices (RIs) with the occurrence of surgical complications and allograft outcomes.

**Methods:**

The study included all kidney allograft recipients undergoing transplantation at our center between January 2010 and December 2011 (*N* = 329). Ultrasound examination was performed on 315 recipients (95.7%).

**Results:**

Delayed graft function was more common in subjects with a high RI (≥0.7) than in patients with an RI < 0.7 (47.2 vs. 28.2%; *p* = 0.032). A lack of arterial signal was detected in eight patients (2.5%), of whom five had a vascular complication that required surgical therapy. In 12 patients (3.8%), RI was 1 without any other signs of vascular impairment. Even though such values can be a sign of venous thrombosis, no case was observed in any of these patients.

**Conclusions:**

The results of our study suggest that ultrasound evaluation of the transplanted kidney shortly after transplantation is a valuable tool not only for detecting vascular complications but also as a predictor of graft outcome regarding delayed graft function.

## Introduction

Kidney transplantation (KTX) has become the gold standard for treatment of end-stage renal disease. However, even though continuous improvements in immunosuppressive therapy and surgical technique have led to excellent short-term patient and graft survival rates [1], surgical complications still represent an important cause of graft dysfunction and loss in the very early phase after KTX [2–4].

Even though vascular complications have become uncommon after KTX, arterial or venous thrombosis still represents a substantial risk for graft loss. The overall risk for vascular complication ranges between 2 and 4%, with renal artery occlusion being the most common cause [5, 6]. Given the deleterious consequence of a lack of perfusion, several diagnostic screening methods have been proposed in order to detect this kind of early surgical complication.

Ultrasound is cheap, noninvasive, widely available, and efficient for evaluating the allograft and not only depicting the anatomy of the transplanted kidney but also assessing the vascular supply, which is especially at risk in the early phase after KTX. The intrarenal resistance index (RI) calculated from the pulsatile flow-velocity waveform is one marker assessed routinely. Additionally, perirenal fluid retention can be detected, which may indicate any kind of bleeding or compression of vital structures. Moreover, it has been shown that both early and late RI may be predictive of transplant outcome [7, 8]. Impedance index elevations were observed in various conditions affecting graft function such as rejection, urinary obstruction, and acute tubular necrosis [8]; high RI values are rarely found in normally functioning allografts [9].

However, even though ultrasound of the transplanted kidney is performed in most centers worldwide, the clinical significance of early ultrasound examination is still debated. In this large retrospective study, we sought to investigate whether and to which extent the results of ultrasound examination shortly after surgery are associated with allograft function in the early post-transplant period.

## Patients and methods

All kidney transplant recipients undergoing transplantation between January 2010 and December 2011 at the Medical University of Vienna were included in this retrospective analysis. Overall, 329 patients were analyzed for this study. The study was reviewed and approved by the institutional review board of the Medical University of Vienna (IRB no.: 1541/2013).

### Ultrasound

**Fig. 1:**
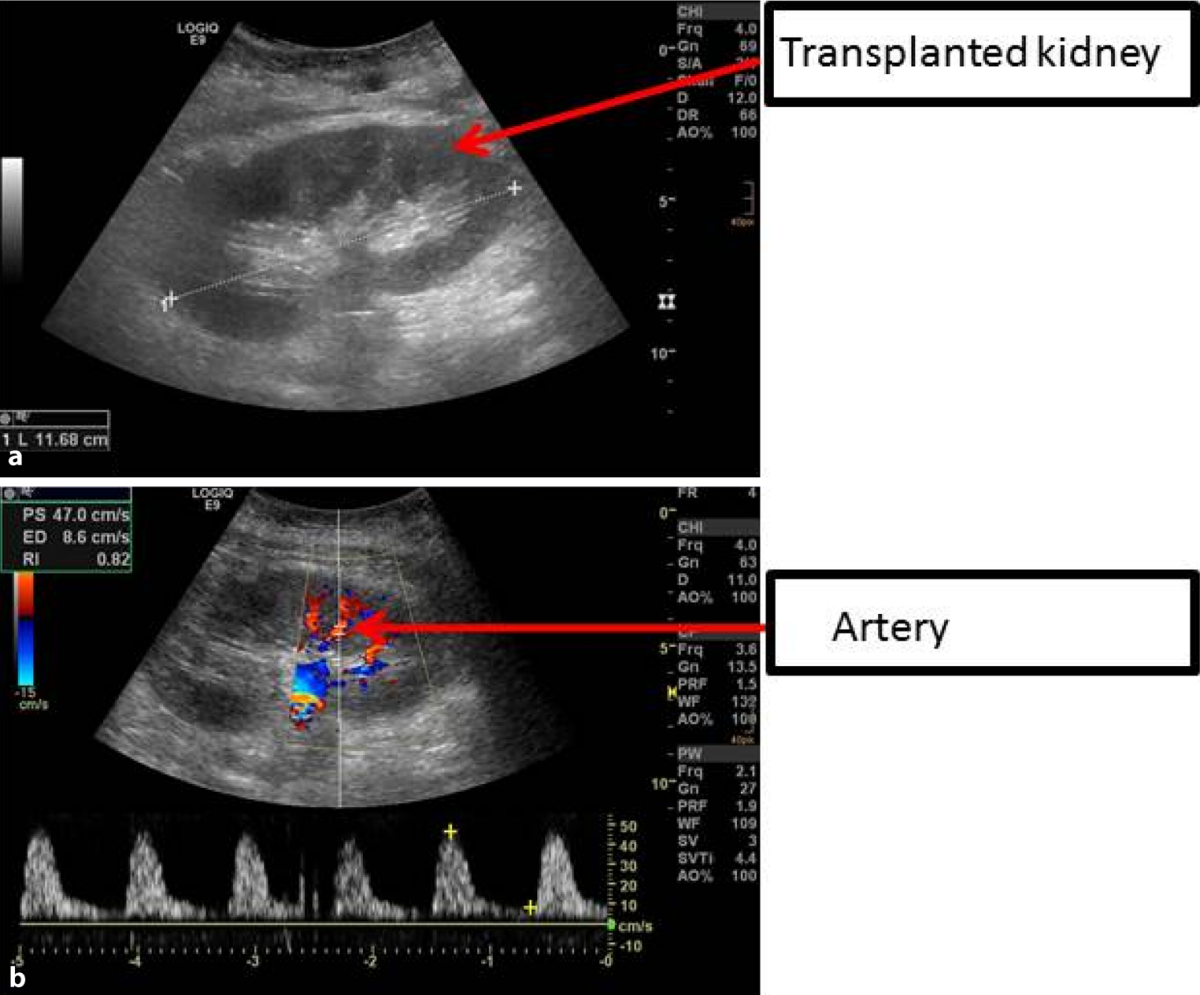
**a** Representative ultrasound image of a transplanted kidney. **b** Normal resistance index is measured in an interlobar artery

Following KTX, ultrasound was routinely performed within the first 48 h of transplantation (Fig. 1). The intrarenal resistance index was calculated as 1‑(Vmin/Vmax) and the results of two or three different measurements were averaged. For survival analysis, patients were divided into high RI (RI ≥ 0.7) and low RI (RI < 0.7) groups [10].

### Endpoints

All data were collected from patients’ charts, laboratory records, and a prospective transplant database. Kidney function was assessed and labeled as delayed graft function (DGF; defined as the need for dialysis for more than 7 days after transplantation), slow graft function (SGF; a serum creatinine equal to or more than 3 mg/ml on the fifth postoperative day) or primary non-function (PNF; a graft that never gained function), as appropriate. Surgical complications were defined as any complications within 3 months associated with the KTX that required surgical therapy.

### Statistical analysis

Statistical analysis was performed using GraphPad Prism, version 5 (GraphPad Prism Software, La Jolla, CA) and SPSS software (version 20.0). Continuous data are expressed as median with range or mean ± standard deviation (SD). Analysis was performed with the Mann–Whitney *U* test or *t* test. Binary outcome variables were compared with Fisher’s exact test and the chi-square test. Furthermore, we used logistic regression models with RI as predictor variable to assess the effect of RI on the studied endpoints. Factors influencing the RI were analyzed using univariate linear regression models. Significant variables were further tested in a multivariate model. The risk of death and graft loss depending on the RI is shown in a Kaplan–Meier graph and analysis was performed by using a log rank test. A *p* value of < 0.05 was considered statistically significant.

## Results

### Patient characteristics

Baseline characteristics are shown in Table 1. Between January 2010 and December 2011, 329 patients underwent KTX at our department and an early ultrasound was performed on 315 patients (95.7%). The study cohort mainly included recipients of a deceased donor allograft (86.3%). The mean recipient age was 51.6 years (16.1).

**Table 1:** Patient characteristics

Patient characteristics	Overall *n* = 315
Sex (female), *n* (%)	117 (37.1)
Recipient age (years), mean (SD)	51.6 (16.1)
Donor age (years), mean (SD)	53.7 (17)
Living donation, *n* (%)	43 (13.7)
*Combined TX, n (%)* SPKT Kidney + liver Kidney + heart Kidney + lung	14 (4.4) 10 (3.2) 2 (0.6) 1 (0.3) 1 (0.3)
Re-transplant, *n* (%)	47 (14.9)
HLA MM, median	3 (1)
CIT (h), mean (SD)	12.3 (6.8)
Sensitized, *n* (%)	21 (6.7)

*TX* transplantation, *SPKT* simultaneous kidney pancreas transplantation, *HLA* human leukocyte antigen, *CIT* cold ischemia time, *MM* mismatch, *SD* standard deviation

### Ultrasonographic findings in the transplanted kidney

**Fig. 2:**
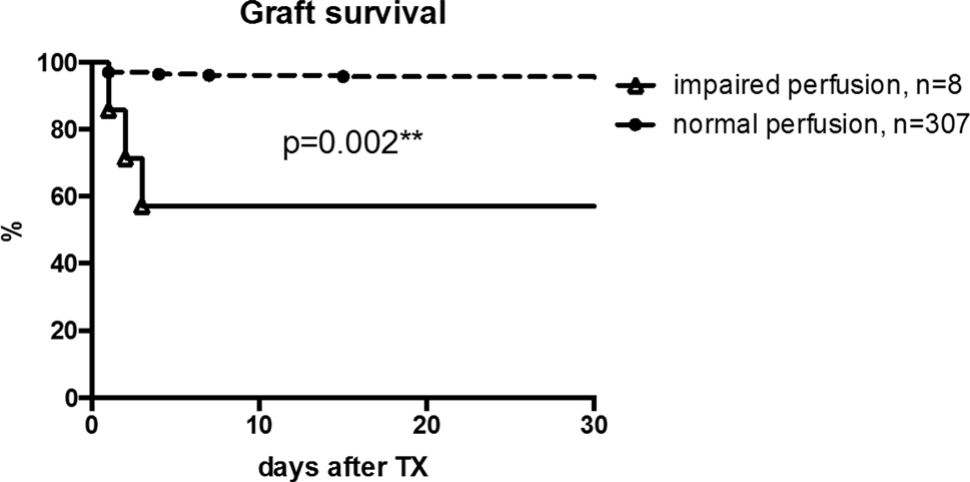
Impaired perfusion during early ultrasound examination was connected to a diminished graft outcome compared with patients without impaired perfusion (*p* = 0.002). *TX* transplantation

In eight patients, a lack of arterial perfusion was noted (2.5%). Vascular thrombosis was confirmed by further diagnostic steps including computed tomography (CT) angiography in five of these cases. After further diagnostic steps including CT angiography, three results were false positive giving a positive predictive value (PPV) of 62.5%. The remaining five patients with confirmed lack of perfusion were scheduled for surgery. There were three patients (1%) showing arterial thrombosis and two patients (0.6%) showing venous thrombosis. Despite immediate surgical intervention, four of these five patients lost their graft. In one patient with arterial thrombosis, successful thrombectomy was performed after flushing the kidney with preservation solution. Overall, patients with a lack of perfusion in the early ultrasound examination had a significantly diminished graft survival compared with patients without (*p* = 0.002; Fig. 2).

### Resistance indices and their impact on outcome

**Fig. 3:**
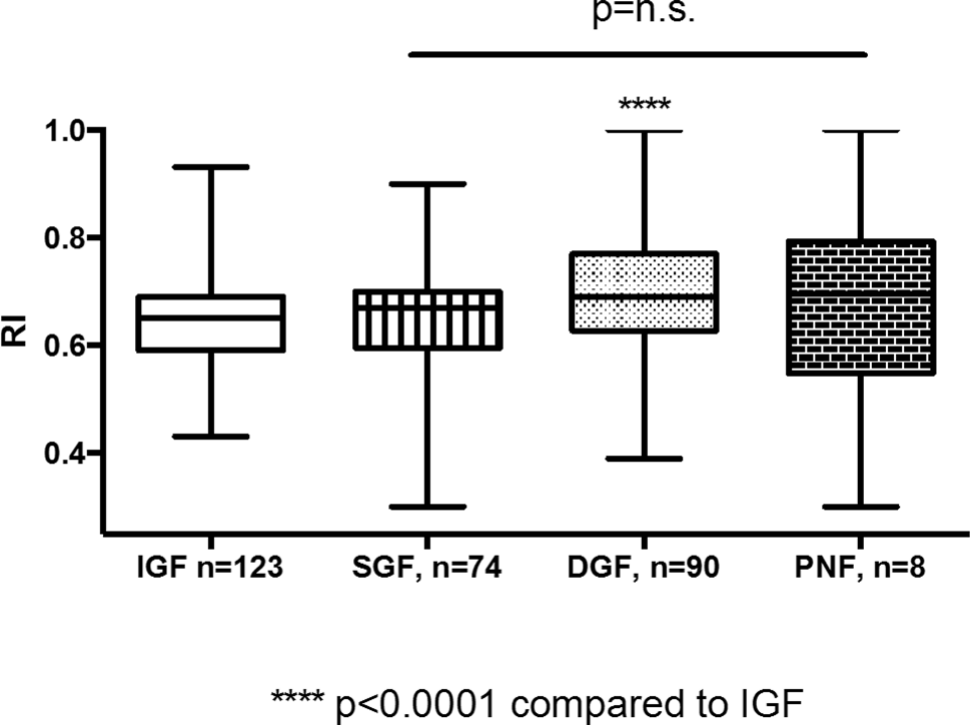
Kidney function. Patients with DGF had significantly higher RI values during early ultrasound examination compared with patients with immediate graft function (*p* < 0.0001). *DGF* delayed graft function, *IGF* immediate graft function, *n.s.* not significant, *PNF* primary non-function, *RI* resistance index, *SGF* slow graft function

Resistance index values were available for 295 patients. Twelve patients had a resistance index of 1, without any other signs of vascular impairment (3.8%). Even though there was a high rate of delayed graft function within this cohort (75%), there was no association with patient or graft survival. In line with these findings, kidney function was significantly impaired at higher RI values. Logistic regression revealed a significantly higher probability of DGF at a higher RI (*p* < 0.0001). Conversely, patients with DGF had a significantly higher RI compared with patients with immediate graft function (IGF; Fig. 3).

**Fig. 4:**
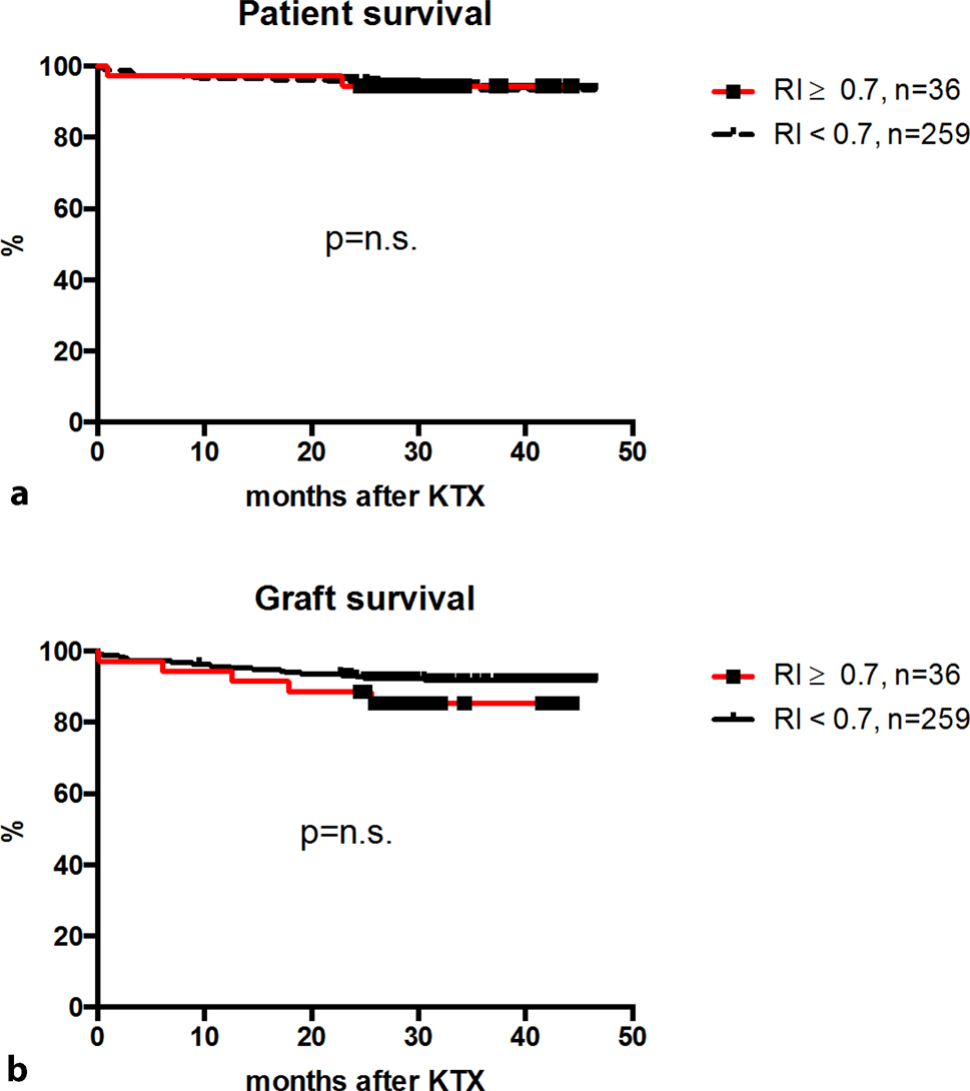
Long-term outcome. There was no significant difference between patients with high (RI ≥ 0.7) or low RI values (RI < 0.7) regarding death-censored graft outcome (**a**) or overall patient survival (**b**). *KTX* kidney transplantation, *n.s.* not significant, *RI* resistance index

However, no association between RI and the incidence of surgical complications (*p* = 0.854) or the incidence of biopsy-confirmed acute rejection (BPAR; *p* = 0.171) was observed. Notably, there was no difference between patient- (*p* = 0.987) or death-censored graft survival (*p* = 0.214) between high or low RI (Fig. 4).

To assess variables influencing RI values, recipient age, donor age, body weight, and cold ischemia time (CIT) were imputed in a univariate linear regression model. Whereas recipient age (*p* < 0.001) and CIT (*p* = 0.016) were significantly associated with vascular resistance, donor age and body weight showed no correlation with RI. These findings were further specified in a multivariate analysis, which showed a significant correlation between RI and recipient age (*p* = 0.001) but not CIT (*p* = 0.072).

## Discussion

In spite of significant progress in surgical techniques over the past decades, complications still represent an important risk factor for early graft or patient loss [3, 4]. One way to improve surgical outcome is an early diagnosis of complications that may arise, in order to treat them in time. Ultrasound examination is a cheap, ready-to-use, and a noninvasive tool, which makes it an ideal candidate as a screening method for patients at high risk after KTX. RI has been evaluated early and late after KTX as a marker for long-term outcome [7, 8].

Graft loss due to arterial or venous thrombosis is one of the most deleterious complications that may arise in the early phase after kidney transplantation. In this study we recorded nine cases of early graft loss (graft loss <14 days after TX), an incidence similar to that described in the literature [5, 6]. Most of these occurred >48 h after surgery and were therefore not detected by early ultrasound examination. The time window to successfully treat a lack of blood supply is narrow, especially in the early phase after transplantation when the kidney is still under stress dealing with ischemia reperfusion injury [11]. Therefore, it is not surprising that most of the grafts with arterial or venous thrombosis were lost despite prompt intervention with emergency surgery. Nevertheless, one graft could be saved by immediate thrombectomy performed after proper diagnosis.

The predictive value of RI has been shown by various authors [7, 8]. We could confirm a significant correlation with RI and early kidney function. It has been suggested that RI values of 1 might be a sign of venous thrombosis [8]. In this study we confirmed the close relationship between kidney function and RI. However, even though patients with an RI value of 1 had a higher probability of developing DGF, there was no case of venous thrombosis in this cohort. Thus, we conclude that elevated RI alone without signs of an impaired perfusion is rare albeit not dangerous.

In conclusion, our results reinforce the value of ultrasound as a diagnostic tool for evaluating the transplanted kidney. In addition to information regarding vascular supply, RI can serve as a fair predictor of outcome.

## References

[1] OPTN/SRTR annual data report. 2012.

[2] Bentas W, Jones J, Karaoguz A (2008). Renal transplantation in the elderly: Surgical complications and outcome with special emphasis on the eurotransplant senior programme. Nephrol Dial Transplant.

[3] Eufrasio P, Moreira P, Parada B (2011). Renal transplantation in recipients over 65 years old. Transplant Proc.

[4] Bessede T, Droupy S, Hammoudi Y (2012). Surgical prevention and management of vascular complications of kidney transplantation. Transpl Int.

[5] Aktas S, Boyvat F, Sevmis S (2011). Analysis of vascular complications after renal transplantation. Transplant Proc.

[6] Dimitroulis D, Bokos J, Zavos G (2009). Vascular complications in renal transplantation: A single-center experience in 1367 renal transplantations and review of the literature. Transplant Proc.

[7] Radermacher J, Mengel M, Ellis S (2003). The renal arterial resistance index and renal allograft survival. N Engl J Med.

[8] Naesens M, Heylen L, Lerut E (2013). Intrarenal resistive index after renal transplantation. N Engl J Med.

[9] Salgado O, Garcia R, Henriquez C (2003). Severely elevated intrarenal arterial impedance and abnormal venous flow pattern in a normal functioning kidney graft. Transplant Proc.

[10] Akgul A, Ibis A, Sezer S (2009). Early assessment of renal resistance index and long-term renal function in renal transplant recipients. Ren Fail.

[11] Chen CC, Chapman WC, Hanto DW (2015). Ischemia-reperfusion injury in kidney transplantation. Front Biosci (Elite Ed).

